# A Microwave Polarimeter Demonstrator for Astronomy with Near-Infra-Red Up-Conversion for Optical Correlation and Detection

**DOI:** 10.3390/s19081870

**Published:** 2019-04-19

**Authors:** Francisco J. Casas, David Ortiz, Beatriz Aja, Luisa de la Fuente, Eduardo Artal, Rubén Ruiz, Jesús M. Mirapeix

**Affiliations:** 1 Instituto de Física de Cantabria (IFCA), Avda. Los Castros s/n, 39005 Santander, Spain; ortizgd@ifca.unican.es; 2 Departamento Ingeniería de Comunicaciones (DICOM), Universidad de Cantabria, Plaza de la Ciencia s/n, 39005 Santander, Spain; beatriz.aja@unican.es (B.A.); luisa.delafuente@unican.es (L.d.l.F.); artale@unican.es (E.A.); 3 Grupo de Ingeniería Fotónica, Universidad de Cantabria, Plaza de la Ciencia s/n, 39005 Santander, Spain; ruben.ruiz@aimen.es (R.R.); jesus.mirapeix@unican.es (J.M.M.); 4 Biomedical Research Networking Center in Bioengineering Biomaterials and Nanomedicine (CIBER-BBN), Plaza de la Ciencia s/n, 39005 Santander, Spain; 5 Instituto de Investigacion Sanitaria Valdecilla (IDIVAL), Calle Cardenal Herrera Oria, 39011 Santander, Spain

**Keywords:** instrumentation, astronomy, polarization, cosmic microwave background, microwave photonics, direct imaging, synthesized imaging, interferometry

## Abstract

This paper presents a 10 to 20 GHz bandwidth microwave polarimeter demonstrator, based on the implementation of a near-infra-red frequency up-conversion stage that allows both the optical correlation, when operating as a synthesized-image interferometer, and signal detection, when operating as a direct-image instrument. The proposed idea is oriented towards the implementation of ultra-sensitive instruments presenting several dozens or even thousands of microwave receivers operating in the lowest bands of the cosmic microwave background. In this work, an electro-optical back-end module replaces the usual microwave detection stage with Mach–Zehnder modulators for the frequency up-conversion, and an optical stage for the signals correlation and detection at near-infra-red wavelengths (1550 nm). As interferometer, the instrument is able to correlate the signals of large-format instruments, while operating as a direct imaging instrument also presents advantages in terms of the possibility of implementing the optical back end by means of photonic integrated circuits to achieve reductions in cost, weight, size, and power consumption. A linearly polarized input wave, with a variable polar angle, is used as a signal source for laboratory tests. The receiver demonstrator has proved its capabilities of being used as a new microwave-photonic polarimeter for the study of the lowest bands of cosmic microwave background.

## 1. Introduction

Penzias and Wilson in 1964 measured a noise-like signal [1] that was finally identified as the cosmic microwave background (CMB). This radiation is the remaining footprint of the Big Bang and was postulated by Gamow, Alpher, and Herman in the late 1940s [2]. CMB intensity and polarization measurements have been an invaluable resource for testing cosmological models and fundamental physics, since the processes that operated in the early Universe, or acted on the photons during their passage to the Earth, have imprinted very weak but distinct features on the otherwise uniform background. Space missions [3,4,5,6] and ground-based experiments [7,8,9] have been dedicated, with increasingly higher sensitivity, to the analysis of temperature and polarization anisotropies of the CMB, with the aim of measuring the B-mode polarization pattern predicted by inflationary models of the early Universe. B-mode signals may reveal crucial information about our Universe, such as if inflation really happened, the signal level of primordial gravitational waves predicted by inflation, the number of neutrino species and their mass, the existence of magnetic fields in the early Universe, and the origin of magnetic fields in galaxies and galaxy clusters. Accurate measurements of B-modes can also reveal (or place limits on) cosmic birefringence, a revolutionary departure from the standard model that allows one to probe the validity of fundamental symmetries, investigate the nature of dark matter, and test extensions of general relativity. B-modes can have different origins, arising from either the primordial gravitational waves produced at inflation, primordial magnetic fields, the gravitational lensing of the CMB, or astrophysical emissions (mainly synchrotron and thermal dust).

B-mode signals are faint and can be easily contaminated. So, only with the use of ultra-sensitive instrumentation is it possible to detect and characterize both B-modes and foreground contamination that appear at different frequencies. The number of receivers or detectors required to measure the B-mode polarization depends on the frequency. The signal from the sky is formed mainly by contaminants as synchrotron and thermal dust, while the CMB represents only a very small fraction of the overall signal. Synchrotron dominates at low frequencies (1–80 GHz) while thermal dust is predominant at high frequencies (400–1000 GHz). Due to this, a lot of the experiments [8,9] designed to measure the B-modes are focused between these two ranges of frequencies (cosmological bands) presenting thousands of detectors (usually bolometers). To remove the contaminants from the CMB signal, it is required to characterize them with accuracy similar to the one of the experiments dedicated to the cosmological bands. At low frequencies, as the ones of the reported demonstrator, the synchrotron emission presents a much higher signal level than the CMB at the cosmological bands. So, in order to have similar accuracy, the required number of detectors or receivers is much lower. In the 10–20 GHz frequency band, an instrument with around 30–40 receivers should be enough, while at 30 or 40 GHz, 300–400 would be needed to characterize the synchrotron with the required accuracy.

Most of the present active experiments operate as direct image telescopes whose number of receivers, and hence, sensitivity, is limited by the space available in the focal plane. This aspect is especially critical when operating at the lowest bands of the microwave range (1–20 GHz), where the size of the receiver horn antennas is bigger. Therefore, alternative ways to achieve better sensitivities must be considered. The use of an interferometer overcomes the space limitation of telescopes and avoids their high cost, by potentially correlating a much larger number (hundreds or even thousands) of receivers or detectors. However, the number of receivers for CMB low-frequency interferometers is typically less than 20, due to the limitations of traditional analog correlators in terms of phase controlling and routing of a high number of wide-band microwave signals. On the other hand, the use of digital correlators does not seem the most suitable option for this type of CMB experiments, due to the high cost (relative to the overall experiment budget) and power consumption of the large number of broadband digitizing cards needed to correlate a large number of wide-band signals in real time and during several months or even years. Other issues, as the requirement of a complex down-conversion and channelization structure and the digitalization noise added to the signals, must also be taken into account. However, for big experiments with as large budgets as the Square Kilometre Array (SKA) [10], all these aspects probably do not represent insurmountable issues. In fact, in that particular case, digital electronics is the technology used to correlate signals from their very high-resolution astronomical observations.

**Figure 1:**
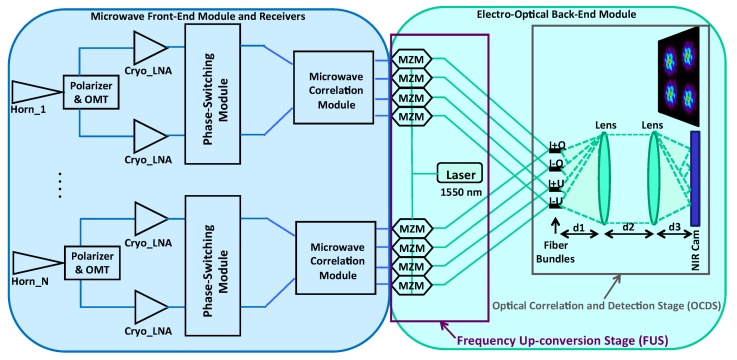
Simplified block-diagram of the microwave polarimeter for N receivers with correlation/detection in the near-infra-red (1550 nm).

The use of electro-optical correlators is an alternative solution already implemented in embedded instruments designed for security and defense [11], and also proposed for astronomy as a Michelson correlator [12]. They allow a drastic reduction in the complexity of correlating the large amount of wideband microwave signals for large-format instruments, in contrast to microwave correlators and, moreover, avoiding expensive telescopes. In this work, this concept is applied to a microwave polarimeter [13] operating in two frequency sub-bands: From 10 to 14 GHz and from 16 to 20 GHz. In particular, a demonstrator is developed to test the operation and viability of the instrumental concept shown in Reference [11], applied to CMB astronomical experiments. Figure 1 shows a simplified block diagram of the polarimeter instrument. The prototype developed in this work is composed of a front-end module (FEM) connected to two microwave receivers, operating in the two sub-bands previously mentioned and an electro-optical back-end module (EOBEM) with a frequency up-conversion stage (FUS) at the input, connected to an optical correlation and detection stage (OCDS). The microwave receivers share the conceptual design of the ones of QUIJOTE experiment [14,15], but some modifications are included to split the 10–20 GHz input signal in two sub-bands of interest (10–14 GHz and 16–20 GHz). On the other hand, the detection stage of QUIJOTE-like receivers is replaced here by the EOBEM with an input microwave to near-infra-red (NIR) FUS, composed of a laser and a set of commercial LiNbO_3_ Mach–Zehnder modulators (MZM), and an OCDS implemented basically with a fiber array, a pair of lenses, and a camera. All these components are already described in Reference [16]. An advantage of this concept is that the same OCDS can be used to operate both as a synthesized-image interferometer and also as a traditional imager, only by changing the optical configuration of the OCDS. In the first case, the instrument provides a synthesized image of the polar parameters in the sky region determined by the instrument field of view (FoV), which is mainly determined by the beam of the horn antennas. In the second case, the OCDS is basically a NIR detection stage of the up-converted microwave signals that have the polar information of the CMB. In the last case, a telescope is required to focus the signal from the sky to the instrument, but the potential implementation of the EOBEM and part of the receivers in narrow-band PIC (photonic integrated circuits) technology, makes this concept also interesting, expecting reductions in cost, volume, weight, and power consumption with respect to wide-band traditional microwave technology.

In this work, a demonstrator of the proposed concept is developed and tested in the laboratory using a linearly polarized input wave with a variable polar angle as excitation. The operation of the polarimeter is tested in both modes of operation (interferometry and direct image). Due to the commercial MZM bandwidth limitations, only the signal from 10 to 12 GHz is characterized, while the rest of the setup allows for the measurement of the complete two sub-bands of interest (10–14 GHz and 16–20 GHz).

This document is divided into five sections. The first one is an introduction, followed by a detailed description of the proposed polarimeter in Section 2. In Section 3, the polarimeter operation is presented showing laboratory measurement results. The demonstrator possible enhancements, potential, and future plans are discussed in Section 4, and, finally, Section 5 draws the general conclusions from this work.

## 2. Polarimeter Description

In this section, the microwave polarimeter, shown in Figure 1 for N receivers, is described. As mentioned previously, the instrument is composed of a microwave FEM connected to receivers and an electro-optical back-end module with the FUS at the input, connected to an OCDS. As it is shown in Section 2.2, the dual operation of the polarimeter is determined by the distances between the fiber array, the lenses, and the camera (d_1_, d_2_, and d_3_). Due to the particular design of the microwave receivers, the four output signals of the microwave correlation module are proportional to a combination of the Stokes parameters (I + Q, I − Q, I + U, I − U). Their values are determined by the polarization of the input wave [17,18]. That is the reason for having four fiber arrays (bundles) and four images represented in the NIR camera.

The reported demonstrator is down-scoped with respect to the polarimeter scheme shown in Figure 1. First, the number of receivers (N) is only two, which is the minimum number required to be used as an interferometer. The operation of the two-receiver demonstrator is tested in direct image and also in interferometry operation mode. Only one fiber array with 46 fibers is implemented for the demonstrator so the interferometer mode of operation is tested by correlating two signals, each one coming from the corresponding two receivers set of four output signals. Therefore, only one synthesized image, providing the complete polar information of the input wave, is obtained in the interferometry tests, as it is explained in References [17,18] and described in the next sections.

### 2.1. Front-End and Microwave Receivers

**Figure 2:**
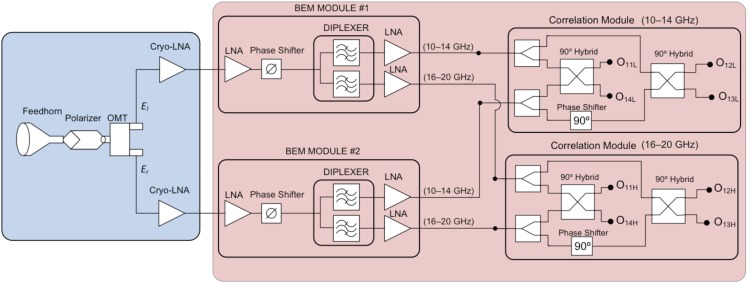
Detailed schematic of the front-end and microwave receivers designed for this work.

In this section, some details about the microwave receivers are explained. As it was mentioned before, the receivers have the same conceptual design of the ones of QUIJOTE experiment [14,15] but with some improvements and optimization in the design. In particular, the operation bandwidth is the same of the QUIJOTE multi-frequency instrument (MFI) [15], but with a receiver design using an electronic polarization modulation similar to thirty- and forty-gigahertz instruments (TGI and FGI) [19]. On the other hand, while MFI has two polarimeters covering the band from 10 to 14 GHz and the other two covering the band from 16 to 20 GHz, the receivers of the proposed demonstrator split the full 10–20 GHz input signal to obtain the Stokes parameters of the two sub-bands in each polarimeter. Therefore, it would be possible to optimize the filling of the telescope focal plane, improving the sensitivity of the resulting instrument. Figure 2 shows a detailed diagram of the front-end and microwave receiver implemented for this work. Front-end and first back-end elements are designed to cover the overall 10–20 GHz bandwidth. Then, the diplexer splits the original bandwidth in the two sub-bands of interest (10–14 GHz and 16–20 GHz) to feed two correlation modules, which combine the signals in such a way that, at the output, the microwave signals are proportional to a combination of the corresponding Stokes parameters in each frequency band.

**Figure 3:**
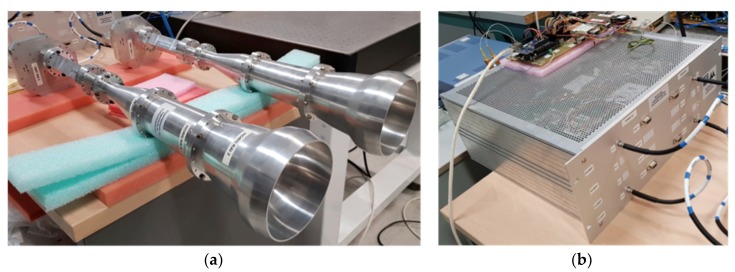
(**a**) Picture of the two feed-horns, orthomode transducer (OMT), and polarizers assembled together. (**b**) Picture of the rack containing two units of the ambient-temperature microwave receivers of Figure 2.

In Figure 2, the cryogenic microwave front-end (colored in blue) is cooled-down to 20 K in order to have the required signal to noise ratio when making astronomical observations of the CMB. It is comprised of a feed-horn, a polarizer, and an orthomode transducer (OMT) to split the polar components of the signal, which are introduced into two cryogenic low noise amplifiers (LNA) [17,20]. All these components cover the overall bandwidth from 10 to 20 GHz. Figure 3a shows pictures of the passive components of the microwave front-end.

Also, in Figure 2, the ambient-temperature microwave receiver (colored in red) is comprised of two gain, polarization modulation (phase-switching), and diplexer input modules (cited as BEM modules in Figure 2), followed by two microwave correlation output modules. As the aim of this work is to demonstrate the operation of the proposed instrument concept, the prototype implemented and tested in the laboratory for this work is an ambient-temperature version of the one shown in Figure 2, removing only the cryogenic LNA and without using a cryostat. Consequently, the laboratory polar test signal is generated with the required power to be detectable, presenting a higher power level than the noise added by the receiver itself [20]. Figure 3b shows a picture of a rack containing two units of the ambient-temperature microwave receivers of Figure 2 assembled and operating in the laboratory. Over the rack, a PCB with an Arduino system that is used to control the polarization modulation stage can be seen, composed of the two phase-switches also shown in Figure 2. These phase-switches are composed of two commercial units, CGY2173UH from OMMIC, which is a high-performance GaAs MMIC 6–bit phase shifter operating nominally from 6 GHz up to 18 GHz but working quite well until 20 GHz. The CGY2173UH has a nominal phase shifting range of 0°–360° in 5.625° steps (64 phase-states). For our tests, only four different phase-states are used by shifting in 90° steps [17,18]. The required 6-bits control signals are provided by the previously mentioned Arduino system.

### 2.2. Electro-Optical Back-End

As it is shown in Figure 1, the electro-optical back-end is composed of the input frequency up-conversion stage (FUS) connected to the OCDS, which presents two different optical configurations depending on the type of operation (interferometer/imager). The demonstrator of this work has only two receivers, which is the minimum number required to be used as an interferometer. The commercial Mach–Zehnder modulators (MZM) used in the FUS have a nominal bandwidth of 10 GHz. Hence, only four MZM are selected from the initial set of 16 units tested in the laboratory showing an acceptable response to microwave signals up to 12 GHz with enough level of stability as well as optical carrier rejection [16]. Another difference between the demonstrator and the scheme in Figure 1 is that only one fiber array with 46 fibers is implemented instead of the four shown in that figure. Nevertheless, this is not a limitation to test the operation of the demonstrator, since only four fibers of the array are needed to test the imaging operation of each receiver (one per MZM of the set of four connected to the microwave receiver outputs) and a minimum of only two fibers are needed to test the operation as interferometer. In this last case, the synthesized image resulting from the interference of two up-converted output signals from each receiver is achieved, which corresponds to the same Stokes parameter combination (same output of each receiver).

**Figure 4:**
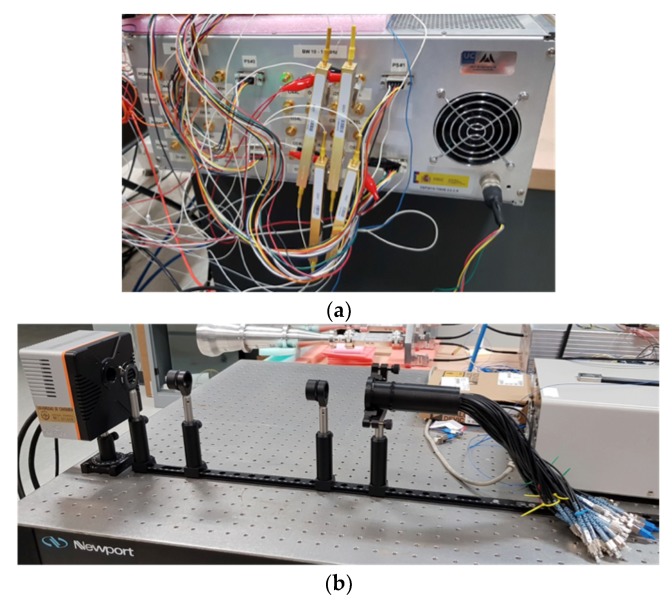
(**a**) Picture of the frequency up-conversion stage (FUS) composed of a set of four Mach–Zehnder modulators (MZM). (**b**) Picture of optical correlation and detection stage (OCDS) composed of the fiber array two lenses and a near-infra-red (NIR) camera.

Figure 4a,b shows pictures of the MZM connected to the rear panel of the microwave receiver rack and the OCDS. Figure 4b shows, from right to left, the fiber array, two lenses of 10 cm focal length, an iris, and the near-infra-red (NIR) optical camera. Most of the components of the OCDS, except the camera, are described in Reference [16].

In this work, the demonstrator is implemented with a 640 × 512-pixel resolution camera from Xenics (Leuven, Belgium), model Cheetah-640-CL, which presents a pixel size of 20 × 20 μm, a spectral band from 0.9 to 1.7 μm, and a frame rate at maximum resolution of 865 Hz. This last characteristic is very important for the reported application, since it is required to acquire the images at a higher rate than the one given by the phase-switching frequency of the microwave receivers. Since the 1/f knee frequency of the cryogenic LNA is expected to be around 10 Hz, the phase-switching rate in real operation should be about 100 Hz to avoid the system gain variation. Therefore, a frame rate of about 1 kHz, which can be easily achieved by slightly reducing the actual acquisition resolution of the camera, is adequate for this particular application. The polarimeter operation mode is determined by the distances between the fiber array, the lenses, and the camera (d_1_, d_2_, and d_3_ in Figure 1) [21]. *f* being the focal length of the lenses, in order to operate as an imaging instrument, a 4*f* optical configuration is used. In this case, d_1_ = d_3_ = *f* and d_2_ = 2*f*. On the other hand, in order to operate as a synthesized image interferometer, a 6*f* optical configuration is used, with d_1_ = 2*f*, d_2_ = 3*f* and d_3_ = *f*. This last case is the one shown in Figure 4b. The reported optical configurations, using two lenses, are well intended for the use of a transmission optical filter to remove the optical carrier and one of the side bands from the modulated NIR signal at MZM outputs. However, the reported demonstrator does not implement such a filter, because an optimal filtering solution operating in transmission has not been found yet. As it is explained in Section 4, the use of a reflection filter is tested instead. Nevertheless, by using the optimal operation point of the MZM and a stabilization method [16], it is possible to demonstrate the instrumental concept in both configurations, as the next section describes.

## 3. Polarimeter Operation

**Figure 5:**
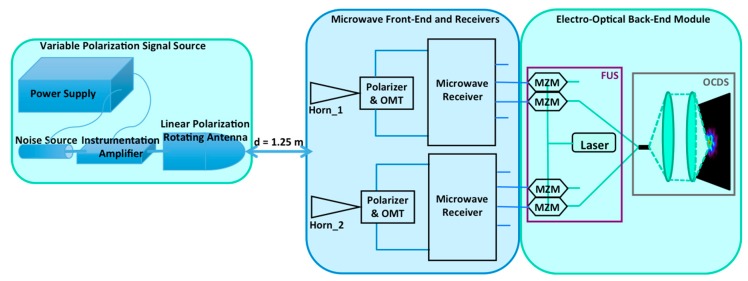
Sketch of measurement test-bench implemented to test the polarimeter demonstrator.

This section is focused on the tests of the polarimeter in both modes of operation. Figure 5 shows a sketch of the measurement test bench implemented in the laboratory. In order to test the polarimeter, a variable polarization source is used, which provides microwave signals in the 10 to 20 GHz frequency range. They are generated with a noise source 346B from Agilent (now Keysight Technologies, Santa Rosa, CA, USA) with excess noise ratio (ENR) 15 dB, amplified with a broadband microwave system amplifier HP83017A of gain 37 dB and noise figure 5 dB at 12 GHz, and transmitted through a broadband log-periodic antenna HL050 from Rohde and Schwarz (Munich, Germany) with almost rotation-symmetrical pattern. The amplifier and the antenna are connected with a coaxial rotary joint, allowing the rotation of the antenna and the generation of polarized signals with different polar angles. As the polar angle variation is implemented by means of a simple hand-controlled mechanical system, the estimated polar angle error of the source is around one degree. In order to test the demonstrator operation this error level is feasible, but for astronomical measurements, a software control system providing lower angular error is required. The far-field distance considering the frequency and antenna diameter is 1.25 m.

**Figure 6:**
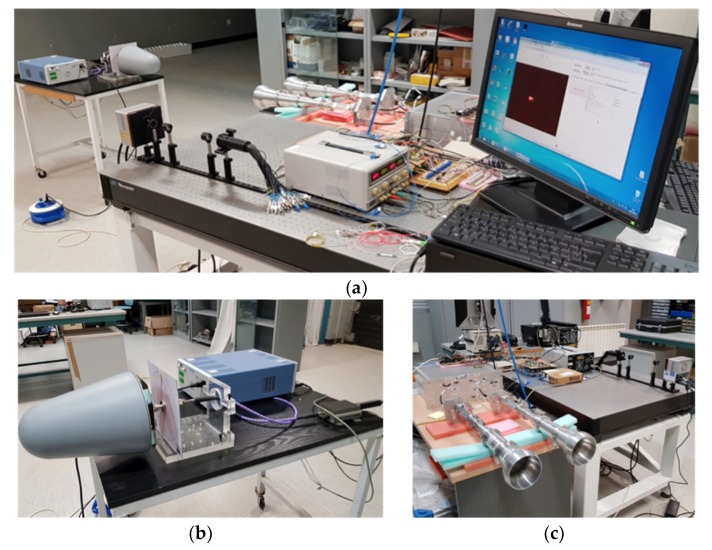
Pictures of the measurement test bench. Overall system operating in direct imaging mode (**a**). Polarized signal source (**b**). Microwave front-end and receivers at forefront (**c**).

This distance is required only for the operation as interferometer (for direct imaging the source could be closer) but in order to simplify the setup, the same distance for the two operation modes is used. Consequently, the power budget is calculated to avoid saturation in the polarimeter receivers. Figure 6 shows some pictures of the measurement test-bench in the laboratory, operating in direct imaging mode (4*f* optical configuration). The measurement procedure in both operation modes is similar, because an imaging instrument performs the polarization measurement. For direct imaging, the power of each receiver is up-converted and detected directly by the NIR camera. For interferometry, the optical system provides the synthesized image that is also detected with the camera. In order to have the same optical power level coming out of each fiber of the bundle, the power level is tuned using variable attenuators. The homogeneity in optical power is required in both operation modes, but it is more critical when operating as an interferometer. In this last case, a tuning method for the phase is also required for instrument calibration.

**Figure 7:**
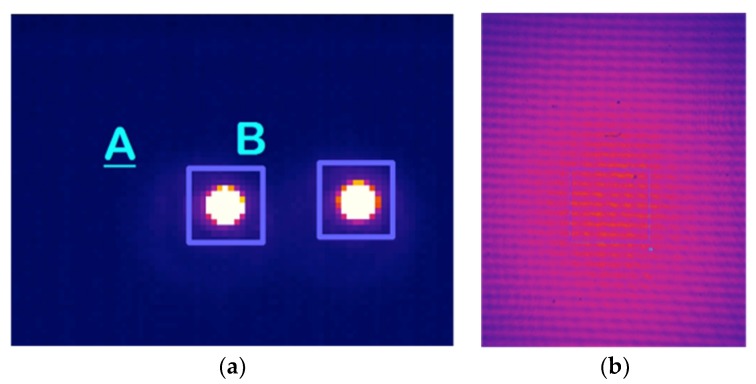
Pictures taken from the camera. (**a**) Two up-converted receiver output signals operating in direct imaging. The mean amplitude value in 14 × 14 pixels is taken as detected signal. (**b**) Synthesized image fringes from the interference of two receiver up-converted output signals. The mean amplitude value in 140 × 150 pixels is taken as detected signal.

The image synthesized by only two receivers is basically a group of fringes, which is not good enough to be used in actual observations. In order to have a cleaner image, more receivers placed in an optimized configuration should be used to provide an optimal point spread function (PSF) (see References [11,16,21] for more details). On the other hand, the polarization measurement method is basically the same that the one explained in References [17,18] for the QUIJOTE experiment. For the measurement tests, the camera acquisition ratio (frame rate) is set to 100 Hz. The applied polarization modulation phase-switching frequency is 3 Hz, so the complete cycle with 16 phase -states provided by the phase switches (four sequences of 0, 90, 180, and 270 deg. phase-shift between receiver’s branches [17,18]) is repeated approximately every 5.4 s. In order to integrate the power of the Near-Infra-Red signals imaged with the camera, the detected signal level is calculated as the mean value in a certain number of camera pixels, where the up-converted signals are imaged (direct imaging) or the maximum of the synthesized image power is found (interferometry). Figure 7 shows two pictures of the images achieved when operating in both modes and the pixel areas selected to measure the average signal level.

### 3.1. Operation as a Direct Imaging Instrument

In this section, the polarimeter demonstrator measurement results when operating in direct image mode is described. The polarized signal source, shown in Figure 6b, is used to generate 100% linearly polarized signals with polar angles between 0° and 180° in 10° step. For each polar angle, a complete sequence of 16 phase-states, provided by the phase switches in the microwave receivers, is used to characterize the amplitude and phase of the input signal polarization vector.

**Figure 8:**
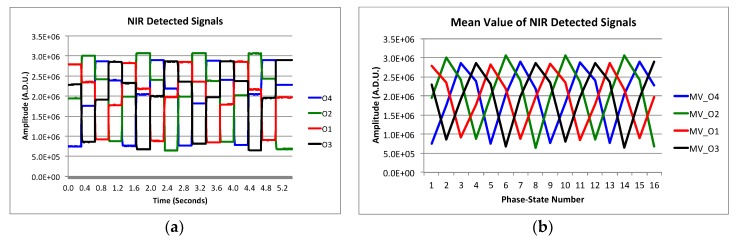
(**a**) NIR detected signal levels with electronically polarization modulation using the phase-switching stage of the receivers. (**b**) Resulting waveform from each phase-state mean value.

**Figure 9:**
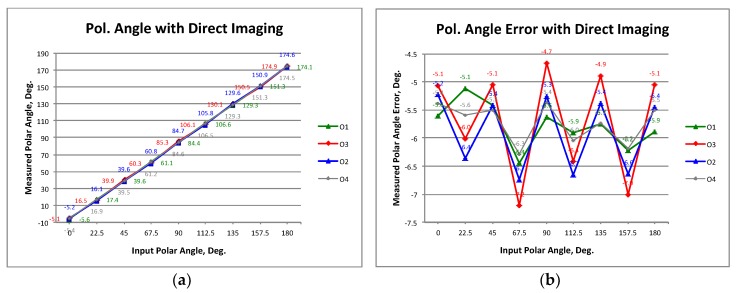

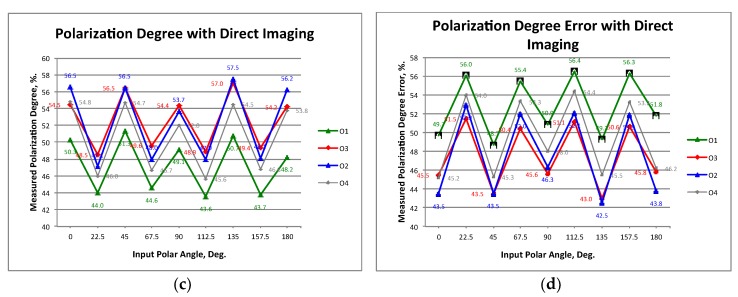
Polarization measurement results of the demonstrator: Polar angles (**a**) and errors (**b**). Polarization degree (**c**) and errors (**d**) calculated with respect to a 100% polarized wave.

Figure 8a shows the resulting NIR detected signal levels of one receiver, for a 0° input polar angle, taking the mean values in square pixel areas as the one shown in Figure 7a. For each phase-state, the mean value of the detected signals is calculated, and waveforms are shown in Figure 8b. The signal level values are given in A.D.U. (A/D converter units of the camera). The amplitude and phase of the polarization vector is extracted from the waveform fundamental harmonic calculated through an FFT [18]. With this procedure, it is possible to measure the polarization of the incoming signal, achieving four values of polar angle and polarization percentage for each receiver. The two receivers of the demonstrator tested in the laboratory show very similar results, therefore only the results of one of them are presented. Figure 9a–d shows the measured polar angles and their errors, and also the achieved polarization percentages (or degree) and their errors for each one of the four detected signals.

It can be seen that the polar angle errors are around 6° and the polarization degree errors present values around 50%. Clearly, the most important error is in the polarization degree so in the next section, the source of these errors and some instrumental modification proposals to reduce them are discussed. Nevertheless, a calibration methodology [22,23] applied specifically to this kind of instrumentation can reduce the systematic errors to the levels required by the new cosmic microwave background (CMB) experiments. The calibration method is based on polar vector error fitting as a function of the measured polar angle, to remove that error directly from the observed polar vectors. The method will be explained in detail in a future work.

### 3.2. Operation as a Synthesized Imaging Interferometer

**Figure 10:**
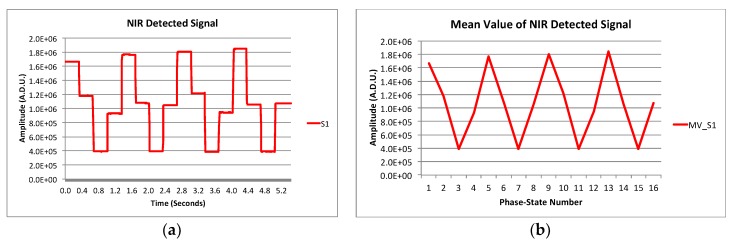
(**a**) NIR detected signal levels with polarization modulation given by the phase-switching stage of the receivers. (**b**) Resulting waveform from each phase-state mean value.

The measurement setup when operating in synthesized image mode uses the same polarized test signal as in the previous section with 1.25 m far-field distance. The polarization modulation is also similar except for the particularity of modulating the polarization synchronously with the two receivers of the demonstrator. One important difference with the direct imaging instrument, in which each receiver is used to obtain independent measurements of the polarization (requiring the use of a telescope), is that now four synthesized images can be achieved in the NIR, making interference of the four pairs of up-converted signals in the OCDS. The operation of the instrument concept is demonstrated using only one of the four possible synthesized images. Figure 10a shows the resulting NIR detected signal levels, for a 0° input polar angle, taken the mean values in the selected region in Figure 7b. For each phase-state, the mean value of the signals is calculated, achieving the waveform of Figure 10b.

**Figure 11:**
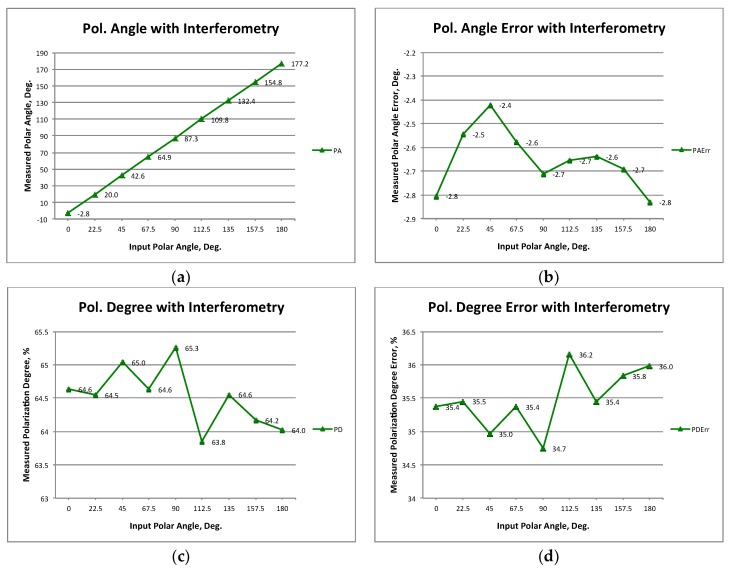
Polarization measurement results of the demonstrator operating as a synthesized image interferometer: Polar angles (**a**) and errors (**b**). Polarization degree (**c**) and errors (**d**) calculated with respect to a 100% polarized wave.

Again, the amplitude and phase of the polarization vector are extracted as in direct imaging operation mode. Figure 11a–d shows the measured polar angles and their errors, and also the achieved polarization percentages and their errors. It can be seen that the polar angle errors and the polarization degree errors are around −2.6 degrees and 35.5%, respectively. It is noticed that the errors are lower than in the direct image operation mode, and this is one of the most important advantages of this type of operation with respect to the previous one. Nevertheless, the polarization degree error is still important, but, as in the previous case, a calibration methodology with specific application to this kind of instrumentation needs to be applied.

## 4. Discussion

In the previous section, the polarimeter operation results are shown when measuring the polarization of a 10–20 GHz bandwidth microwave signal in two modes of operation—direct imaging and synthesized image interferometry. In terms of systematics, the reported design has the advantages given by the typical interferometry such as reduced sensitivity to atmospheric variations, no optical aberrations on the edge of receiver array, or lower sensitivity to beam ellipticity, but presents an operation mode similar to the typical imagers. Additional systematics of the reported system come mainly from the fiber dependence of temperature variations and misalignments of the fiber array that provides errors in the homothetic mapping between microwave receivers and optical fibers. All these issues impact the relative phase between signals, which is a fundamental factor for the image synthesis; therefore, a phase tuning and controlling system must be implemented to operate as a practical interferometer. Temperature control systems and variable phase shifters can be included in the optical chains to control this kind of systematics. However, for the case of direct imaging operation, this is not an important issue, since the optical system basically acts as signal detection stage. Hence, the instrument would be affected by the typical imager systematic errors such as optical aberrations from telescope and atmosphere and gain variations from 1/f noise. On the other hand, the frequency up-conversion performed by the MZM also provides additional systematics due to the optical carrier and additional lateral band of the up-converted signal. The optical carrier can be assumed as an interfering signal that limits the instrument sensitivity and also the polarization efficiency (errors in the measured polarization degree). The lateral band, while in direct imaging operation mode is not an issue, in interferometry operation mode provides optical aberrations and lateral lobes to the synthesized image. These issues can be solved with the use of an optical band-pass filter and also by setting the optimal operation point of the MZM. A thermal control system for the stabilization of the MZM operation point is commonly used in typical astronomical instruments. In our particular case, a feedback technique is also applied to provide the required operation point stability.

### 4.1. MZM Bandwidth

The measured amplitude (polarization percentage or degree) and phase (polar angle) systematic errors of the instrument are shown in Figure 9 and Figure 11, respectively. Taking into account that the polar angle error of the input signal source is around 1°, the measured instrumental phase error does not seem very significant. However, the polarization degree error seems important as only half of the signal polarization is detected in direct imaging while something more is detected with interferometry. The reason for that could come from the loss of signal introduced by the Mach–Zehnder electro-optical modulators (MZM) implementing the frequency up-conversion stage (FUS). The frequency up-conversion from microwaves to the near-infra-red allows for the operation of wide-band microwave signals, as they were narrow-band. Then, technically, the main bandwidth limitation of the proposed instrumental concept would come from the microwave receivers and the MZM. In the reported demonstrator, the bandwidth is limited by the low-cost 10 GHz nominal bandwidth MZM used to implement the frequency up-conversion stage. These MZM provide very important losses for signals from 10 to 14 GHz and are too high for 16 to 20 GHz, and only a frequency band from 10 to 12 GHz presents the required level to be distinguished from the noise floor determined mainly by the not-rejected optical carrier [16]. In order to overcome this issue, higher bandwidth MZM are commercially available to cover bandwidths of more than 100 GHz [24]. However, due to the cost of those MZM, an instrument with hundreds of receivers, as required, for instance, at 30 or 40 GHz, would be unaffordable. On the other hand, since at the frequency band of the demonstrator (10–20 GHz) the required number of receivers is an order of magnitude lower, the economic viability of such an instrument is improved. The development of wide-band MZM implemented with technologies, such as InP or Si, allowing the integration of several units in one compact module, is probably the most viable solutions in terms of cost and performance. Additionally, the integration of the microwave receiver functions in optical technology could optimize the design and reduce the number of required MZM.

### 4.2. Optical Carrier Rejection

**Figure 12:**
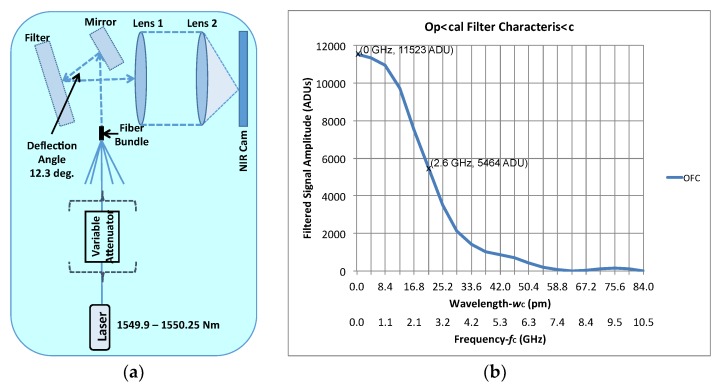
(**a**) Sketch of the measurement test bench implemented to test the optical filter. (**b**) Optical characteristic measured at Instituto de Astrofísica de Canarias (IAC) laboratory as a function of the wavelength and frequency offsets respect to *w*_C_ and *f*_C_.

In the reported demonstrator, a carrier rejection optical filter has not been included for simplicity and because, with only two receivers, it is not possible to achieve a good quality synthesized image (only a group of fringes can be obtained). However, for a general case with a higher number of receivers, the use of optical filters to reject the undesired part of the modulated optical signals is required, not only to improve the polarization degree characterization, but also to be able to generate synthesized images with the required quality to be used in the actual CMB experiments. Figure 12a shows a sketch of the measurement test bench implemented to test a volume bragg optical filter (VBF) at “Instituto de Astrofísica de Canarias” (IAC) laboratory. It can be seen that the filter operates in reflection with a nominal deflection angle that can be slightly modified to tune the filter central wavelength (*w*_C_) or frequency (*f*_C_).

In Figure 12b, the measured filter characteristic is shown. The amplitude of the filtered signal is represented as a function of the wavelength offset with *w*_C_ as a reference. The corresponding values of frequency offset are added to the *x*-axis of the graph to show that the 3-dB bandwidth of the filter is around 5 GHz (40 pm), resulting in enough selectivity for this particular application.

### 4.3. Synthesized Beam

**Figure 13:**
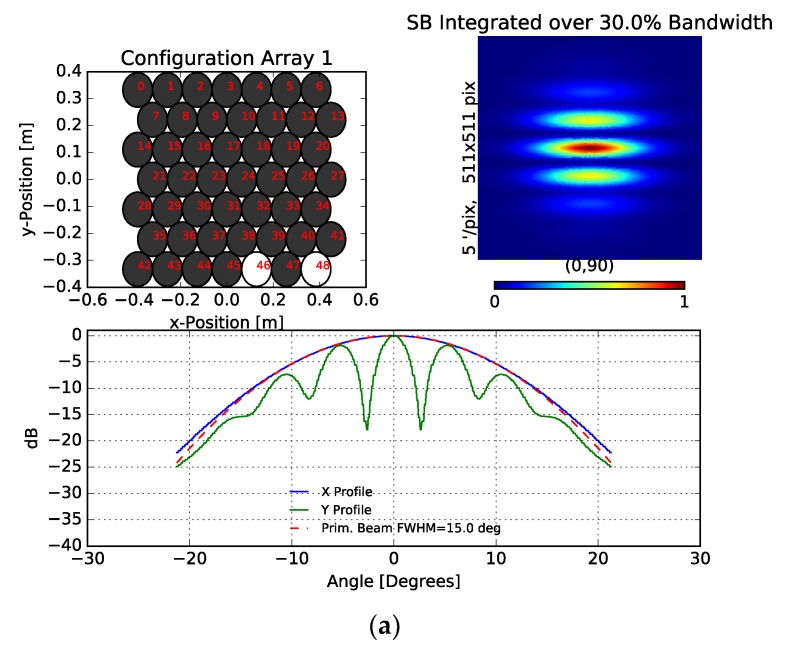

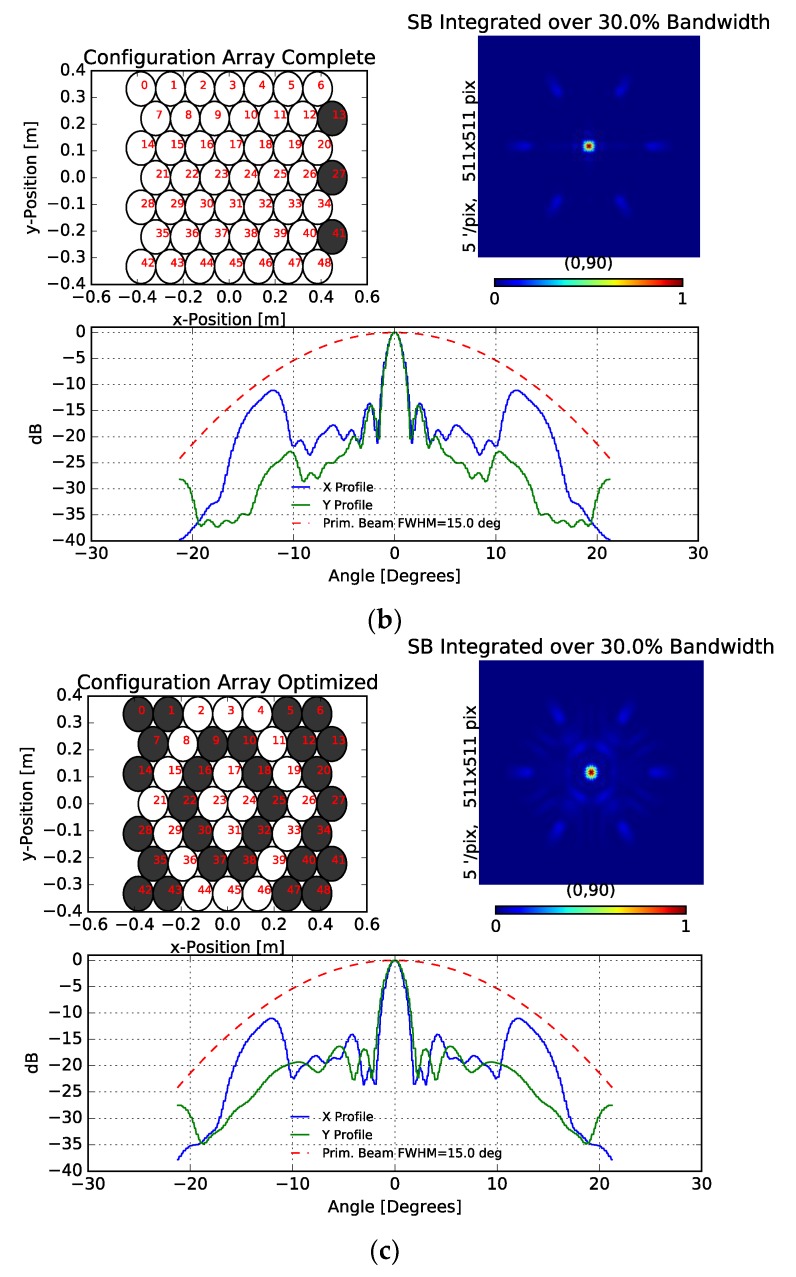
Synthesized beam simulation results. White circles of top-left panels represent active receiver horn antennas and bottom panel shows the X and Y profiles of the synthesized beam (SB) shown in top-right panel. Two-receiver configuration of the reported demonstrator (**a**); fully populated array configuration with 46 receivers (**b**); optimized array configuration with 20 receivers (**c**).

The most relevant additional factors influencing the quality of the synthesized image are the number and distribution of the receivers and the correct homothetic mapping between the horns and the fiber array. The homothetic mapping refers to the common geometry of the antenna and fiber arrays being related by a simple scaling factor. The similarity of both geometries is not perfect due to some misalignments in the fiber array. An antenna mechanical support precisely reproducing the fiber array configuration can be implemented to enhance this situation. Finally, regarding the number and distribution of receivers, Figure 13 shows synthesized beam (SB) simulation results.

Figure 13a shows the simulation results of the demonstrator configuration with only two receivers. In that case, the synthesized beam (SB) presents a group of fringes similar to that achieved with the laboratory measurements. The difference in the power of the fringes for angles higher than 10 degrees are due to the used primary beam model that does not perfectly fit the actual beam of the horns at those angles. Additionally, the number of fringes achieved by simulation is lower than that achieved in the laboratory because of the lack of the optical filter and the resulting interference between both side bands of the modulated signals [25]. On the other hand, simulating a situation with a fully populated array of 46 receivers (see Figure 13b), the SB presents better optical characteristics, since the side lobes are low and the resulting alias free field of view and resolution are similar to the ones required in experiments as QUIJOTE (around 15). However, the number of receivers can be reduced using an optimized array configuration as the one shown in Figure 13c, where 20 receivers form a SB with similar characteristics to the fully populated array one. In any case, one disadvantage of the interferometer concept is that the large angular scales cannot be well characterized [26]; therefore, the synthesized images lose the information from those scales.

## 5. Conclusions

In this work, it is shown that the performance of a microwave polarimeter demonstrator based on the implementation of a near-infra-red frequency up-conversion stage allowing both optical correlation, to operate as a synthesized-image interferometer, and signal detection, to operate as a direct-image instrument. The demonstrator is a down-scoped version of a large-format instrument, with tens to thousands of receivers, to be applied in low-frequency CMB polarization experiments. The demonstrator has only two receivers, which are enough to prove the interferometer concept, but on the other hand, the sensitivity and quality of the achieved synthesized images are not enough to be used as an interferometer in actual CMB experiments. Nevertheless, the results achieved in the laboratory show promising performance in both operation forms. The polar angle errors of the demonstrator are around 6° in direct imaging operation mode and around 2.6° in interferometry operation mode. The obtained error values are not an issue for the proposed application, taking into account that the source polar angle error is 1° and that the measured errors can be corrected through a dedicated calibration method. The highest-level systematic error characterized in the laboratory is the polarization percentage or degree. While in direct image the error values are around 50%, in interferometry, the error values improve until around a 30%. The main factor behind the reported performance is the reduced bandwidth of the electro-optical modulators implementing the frequency up-conversion stage. In order to solve this issue, higher bandwidth modulators could be used to implement an optimized version of that stage, expecting such significant error reductions. Other improvements can be applied, such as the addition of an optical filter to the demonstrator to get better rejection of the optical carrier and lateral band from modulated signals, or the improvement of the homothetic mapping between the receiver horns and the fiber array. The optical quality of the polarization parameter images is expected to be better with the use of a direct imaging instrument assembled in the focal plane of a telescope mainly because of the side lobes of the synthesized beam and the loss of large angular scales that is produced when operating as an interferometer. The beam side lobes provided by a telescope are usually lower than −30 dB, which is much better than the levels achieved until now with synthesized interferometry. In this sense, work towards a receiver array configuration presenting an optimal synthetized beam for astronomy applications is ongoing. Additionally, in contrast to direct imaging with telescopes, synthesized image interferometry provides loss of large angular scales and makes it harder to perform mosaicking of astronomical images. However, the systematic errors obtained with interferometry are lower than those obtained with direct imaging, making the reported instrumental concept very interesting to be developed and tested with observations from an astronomical observatory. As a first step, the reported demonstrator could be adapted and installed in one of the QUIJOTE experiment telescopes to demonstrate the performance and reliability of the frequency up-conversion instrumental concept in actual direct image observations.
